# Prospective Longitudinal Study of Dynamics of Human Papillomavirus 6 and 11 Infection in Anogenital Hairs and Eyebrows of Male Patients with Anogenital Warts and Age-Matched Controls

**DOI:** 10.3390/microorganisms12030466

**Published:** 2024-02-25

**Authors:** Vesna Tlaker, Lea Hošnjak, Mateja Kolenc, Tomaž Mark Zorec, Boštjan Luzar, Marko Potočnik, Jovan Miljković, Katja Seme, Mario Poljak

**Affiliations:** 1 Department of Dermatovenereology, University Medical Center, 1000 Ljubljana, Slovenia; vesna.tlaker@gmail.com (V.T.); marko.potocnik@siol.net (M.P.); 2 Institute of Microbiology and Immunology, Faculty of Medicine, University of Ljubljana, 1000 Ljubljana, Slovenia; lea.hosnjak@mf.uni-lj.si (L.H.); katja.seme@mf.uni-lj.si (K.S.); 3 Institute of Pathology, Faculty of Medicine, University of Ljubljana, 1000 Ljubljana, Slovenia; bostjan.luzar@mf.uni-lj.si; 4 Faculty of Medicine, University of Maribor, 2000 Maribor, Slovenia; miljkovicj@icloud.com

**Keywords:** human papillomavirus, HPV, HPV6, HPV11, genetic variants, anogenital warts, persistence, recurrence, prospective study

## Abstract

To better understand the natural history of anogenital warts (AGWs) and the dynamics of HPV6/11 infection in regional hairs, 32 newly diagnosed male patients with AGWs and 32 age-matched healthy controls were closely followed. During enrollment and six follow-up visits (every 2.6 months), 43 AGW tissues and 1232 anogenital and eyebrow hair samples were collected. This is the closest longitudinal monitoring of AGW patients to date. Patients were treated according to standards of care. The HPV6/11 prevalence was 19.9% in the patients’ hair samples (HPV6 B1 in 53.1%) and 0% in the controls. The highest HPV6/11 prevalence was found in pubic hairs (29.0%) and the lowest in eyebrows (7.1%). The odds of having HPV6/11-positive hairs increased with smoking, shaving the anogenital region, and age. A close association between HPV6/11 presence in hairs and clinically visible AGWs was observed. The proportion of patients with visible AGWs and HPV6/11-positive hairs declined during follow-up with similar trends. No particular HPV6/11 variant was linked with an increased AGW recurrence, but the sublineage HPV6 B1 showed significantly higher clearance from hairs. Despite treatment, 78.1% and 62.5% of the AGW patients experienced one and two or more post-initial AGW episodes, respectively. The patients with HPV6/11-positive hairs or visible AGWs at a preceding visit demonstrated substantially higher odds of presenting with visible AGWs at a subsequent visit.

## 1. Introduction

Anogenital warts (AGWs) are common benign tumors that typically present as flesh-colored exophytic lesions on the external genitalia. The etiological agents of AGWs are human papillomaviruses (HPVs), with HPV types 6 and 11 (HPV6/11) causing more than 95% of cases [1,2,3,4,5,6,7,8,9]. AGWs frequently recur, but it is not clear whether this is due to the inadequacy of treatment or to some particular feature(s) of its causative agents [4,8]. All available AGW treatments are nonspecific and do not eradicate HPV infections [1,2]. Because the lifetime prevalence of AGWs is estimated at between 4% and 10% [4,10,11,12,13], their recurrences are a considerable healthcare problem and contribute to emotional distress, psychosocial stigma, a reduced quality of life, and financial burden [14,15,16,17].

Research on AGWs usually relies on the detection of HPV in tissue samples and/or in swabs of the wart’s surface [17,18]. Prior studies have suggested that the recurrence of AGWs may be attributed to latent HPV infection reservoirs in the surrounding epithelium [18] or in nearby anogenital hair follicles [19]. An HPV infection that is confined exclusively to hair follicles and thus is not present on the skin surface may go undiagnosed when testing the skin surface. Consequently, plucked hairs comprising both the follicles and the surface shafts could provide an optimal, convenient, and easily collected clinical specimen for the diagnosis of a latent HPV infection. Such research on latent HPV infections could provide new perspectives on the natural history of various HPV-related conditions, including the recurrence of AGWs [18].

Our pilot cross-sectional study involving 53 male patients revealed that 43.7% of anogenital hair samples from patients with AGWs were positive for the presence of *Alphapapillomaviruses*, which was much higher in comparison to their presence in apparently healthy controls (4.5%) [20]. Notably, in the aforementioned pilot study, the HPV types identified in AGWs and corresponding hairs were congruent at both the HPV-type and genomic variant levels [20]. A similar cross-sectional study from China found a higher incidence of HPV infections in the pubic hair follicles of patients with AGWs compared to healthy men (32.55% vs. 17.21% respectively), with HPV6 and HPV11 being predominantly identified across both groups [21].

To gain a better understanding of the natural history of AGWs and the dynamics of HPV6 and HPV11 infections in regional hairs and eyebrows, including follicles, among men with AGWs, we conducted a prospective study and longitudinally followed a cohort of 32 male patients newly diagnosed with histologically confirmed AGWs (cases) and 32 age-matched healthy male volunteers (controls) for a period of up to 2 years. We aimed to assess (i) the duration of AGW clinical presence and the dynamics of AGW-causing HPV types (HPV persistence, clearance, and recurrence) in hair samples during the ongoing treatment, (ii) the cross-sectional and longitudinal concordance between HPV types and genomic variants present in AGWs and corresponding hair samples, and (iii) whether specific AGW-causing HPV types or genomic variants could be associated with a prolonged persistence or higher AGW recurrence rates. To meet the study aims, more than 1200 hair samples were tested for AGW-causing HPV types, and all the HPV-positive samples were further characterized. With seven scheduled visits and a mean interval of 2.6 months between individual visits, to the best of our knowledge, our study is the closest longitudinal monitoring of patients with AGWs reported to date.

## 2. Materials and Methods

### 2.1. Study Design

This prospective longitudinal investigation of HPV infections in male patients with AGWs and apparently healthy controls was conducted at the Department of Dermatovenereology, University Medical Center, Ljubljana, Slovenia, in accordance with the Declaration of Helsinki. Ethical approval for this study was granted by the National Ethics Committee of the Ministry of Health of the Republic of Slovenia (consent reference 120-21/2016/15, date of approval: 17 July 2018). Written informed consent was obtained from all the study participants.

This study enrolled a total of 32 patients with newly diagnosed AGWs who were monitored across seven visits scheduled 2 months apart. The control group comprised 32 age-matched sexually active healthy male volunteers with no personal history of AGWs and no present history of AGWs in their current sexual partners. The control subjects were sampled at enrollment and, when possible, at additional time points over the subsequent 2-year period.

During each visit, the patients underwent a detailed examination for the presence of AGWs. Tissue samples were collected from any visible AGWs, taking meticulous precautions to prevent cross-contamination between samples, unless the AGWs were too small to obtain appropriate samples. Each tissue sample collected was bisected by using a scalpel; one half was sent for histopathological confirmation of the clinical diagnosis, and the other half was used for HPV determination.

In addition, at each visit (at enrollment and six follow-up visits), hair samples, including follicles, were collected from three anogenital sites: the pubic, scrotal, and perianal regions as well as the eyebrows in both study groups. The sampling was performed by plucking a pool of three to five hairs by using disposable gloves and sterile tweezers.

After hair sampling, the AGW patients received treatment in accordance with standard care protocols, as deemed appropriate by the treating clinician. The treatment options included cryotherapy, electrodessication, local imiquimod, or other topical treatments [22]. Most of the participating AGW patients were treated by using cryotherapy because this is a preferred treatment for AGWs in Slovenia.

### 2.2. DNA Extraction and HPV Testing

Total DNA extraction, from both the AGW tissues and hairs, was performed by using the High Pure PCR Template Preparation Kit (Roche Diagnostics, Mannheim, Germany) [20]. The isolates’ integrity was verified by a real-time polymerase chain reaction (RT-PCR), allowing for the amplification of the 150 bp of the human beta-globin gene [23]. Beta-globin-positive DNA isolates from the AGW tissues were tested for the presence of HPV6 and HPV11 by using the HPV6/11 real-time polymerase chain reaction (RT-PCR) [24], and the HPV6/11-negative AGW samples were further tested for additional HPV types by using a conventional GP5+/6+/68 PCR in combination with the Sanger sequencing of the PCR products, as described previously [23]. After testing the baseline AGW tissue sample and determining the AGW-causing HPV types, the corresponding hair samples of all patients were tested by using the HPV6/11 RT-PCR [24], while the hairs collected from the patients with baseline HPV6/11-negative AGWs were additionally tested by using the conventional GP5+/6+/68 PCR in combination with the Sanger sequencing of the PCR-products [23].

### 2.3. HPV6 and HPV11 Genomic Variant Characterization

The HPV6 and HPV11 variants were determined based on the 960 and 208 bp representative regions for whole-genome-based phylogenetic clustering [25,26] by using newly developed type-specific PCRs. The HPV6 type-specific primers (HPV6-961-bp-FW: 5′-CCAGATGTAATTCCTAAGGTG-3′ in combination with HPV6-961-bp-RW: 5′-GACAATGGAACTGTGGTGTTAC-3′ (1088 bp), and if necessary followed by HPV6-961-bp-FW in combination with HPV6-961-bp-RWs: 5′-TGTCCATAAAAGCCTCATCA-3′ (751 bp) and HPV6-961-bp-FWs: 5′-TTACAATTACATCCTCTGAAACA-3′ in combination with HPV6-961-bp-RW (787 bp)) were designed manually, based on the multiple alignment (mafft v7.453) [27,28] of the HPV6 L2 nucleotide sequences of the 48 most diverse complete HPV6 genomes [25]. Similarly, the multiple alignment of the target region (the partial E2 gene and noncoding region 2) of 78 complete HPV11 genome sequences was used as a base for the design of HPV11 type-specific primers (HPV11-208bp-FW: 5′-TAGCATCTTCAACGTGGCA-3′ and HPV11-208bp-RW: 5′-TGTTAGTACCAGCACAGATGTATAT-3′ (361 bp)). The selected primers’ specificity was subsequently verified by using the BLAST (http://blast.ncbi.nlm.nih.gov/Blast.cgi, accessed on 15 February 2019) and MFEprimer-2.0 (http://mfeprimer.com/docs/mfeprimer-2.0/, accessed on 15 February 2019) web-based services. The HPV6/11 viral-variant PCRs were performed in a Veriti Thermal Cycler (Thermo Fisher Scientific, Wilmington, NC, USA) by using the FastStart High-Fidelity PCR system (Roche Diagnostics, Mannheim, Germany). Briefly, each reaction mixture contained 1 to 5 μL of template DNA (tissues up to 100 ng) or 3 μL of the outer PCR products in the case of the HPV6-positive samples with low DNA concentrations, 2.5 μL of the 10× FastStart High-Fidelity Reaction Buffer (+1.8 mM MgCl_2_), an additional 1.2 mM of MgCl_2_ stock solution (for the outer PCRs), 200 μM of dNTPs, 0.5 μM of each primer, 1.25 U of the FastStart High-Fidelity Enzyme Blend, and water up to 25 μL. The cycling conditions were as follows: 2 min at 95 °C, followed by 40 cycles of 30 s at 95 °C, 30 s at 52 °C, and 1 min (HPV6)/30 s (HPV11) at 72 °C, followed by a final elongation step of 7 min at 72 °C and the cooling of the reaction mixture to 4 °C. The PCR products obtained were viewed on a 2% agarose gel, Sanger sequenced, and analyzed as described previously [25].

Phylogenetic trees used for the determination of HPV6 and HPV11 genomic variants in newly obtained nucleotide sequences were prepared based on the target nucleotide sequence alignments (mafft v7.453) of the reference genomes (HPV6: *n* = 144 [25]; HPV11: *n* = 78; [26]), nucleotide sequences obtained in our previous studies (HPV6: *n* = 15; HPV11: *n* = 9; unpublished data), and isolates obtained in this study (HPV6: *n* = 28; HPV11: *n* = 3) by using the IQtree (2.0-rc1) [29], adopting the K3P+R2 and GTR+G+I model parametrizations for HPV6 and HPV11, respectively. The node-support values were estimated based on the approximate likelihood ratio (aLRT) [30] and Ultrafast bootstrap (UFBootstrap) [31] methods, with 1000 iterations, and by using the Bayes approach [30]. Subsequently, the identification and naming of the HPV6 and HPV11 genomic variant lineages and sublineages was performed as described previously [25,26].

### 2.4. Statistical Analysis

The sociodemographic characteristics of the study participants were compared by using univariate logistic regression. In the analysis of the categorical variables, descriptive statistics were used to summarize the dataset, including the computation of means, ranges, standard deviations, counts, percentages, and proportions to delineate the distribution of the categorical outcomes. To facilitate the inferential analysis, 95% confidence intervals were calculated for proportions, providing a range within which the true population parameter is likely to fall, assuming a 95% level of confidence. The chi-squared test was used to examine the associations and test for independence between categorical variables.

Based on prior experience with similar patient populations, we anticipated irregular attendance and varying intervals between study visits, and we adjusted our statistical analysis for this real-life situation by allowing each patient up to 2 years to complete the seven scheduled study visits.

The recurrence of AGWs was defined as the clinical re-emergence of AGWs after at least one study visit at which the treating physician found no evidence of the disease, and for the analysis of the recurrence rates, we only considered the timeframe for which data were available for all patients. This approach accounts for the possibility of potential recurrences that may have occurred in participants with shorter follow-up times had they remained in the study longer.

The likelihood ratio test examined the link between HPV types in AGWs or hair samples and AGW recurrence. Agreement on the presence of HPV in AGWs and hair samples was assessed by calculating the proportion of patients with concordant results (both the presence or absence) of each HPV lineage or sublineage across the two sample types. The McNemar test was then used to analyze the significance of any association.

The McNemar test also investigated the consistency of the HPV genotype (sub)lineage determined from hair samples at baseline and after 11 months, which was the cross-sectional point where data were available for all participants.

Additionally, the effects of time (measured in months), the anatomical origin of the hair sample, and their interaction on the presence of HPV in hair samples were studied by using mixed model logistic regression. Both time and the sample origin were treated as repeated measures within a random intercept framework, again assuming an autoregressive correlation matrix. In the final phase of our analysis, demographic and lifestyle variables such as age, smoking habits, and shaving practices were included in the regression model to assess their association with HPV detection in the hair samples.

A significance level of 0.05 was used, and analyses were conducted by using SPSS version 26.

## 3. Results

### 3.1. Characteristics of Study Participants and Follow-Up Visits

All the enrolled 32 AGW patients, aged 17 to 66 (mean: 30.8) years old, completed seven visits with a mean 2.6-month interval between two visits and a total follow-up duration of 11 to 25.1 months (mean follow-up: 15.6 months). The 32 enrolled controls, aged 21 to 52 (mean: 30.7) years, had a mean of 2.6 visits, a mean 4.5-month interval between two visits, and a total follow-up duration of 0 to 21.9 months (mean follow-up: 7.3 months). Most participants were heterosexual, in stable relationships, and with <11 lifetime sexual partners. No significant sociodemographic or sexual behavior differences were noted between the AGW patients and controls (Table 1). However, a significant difference in partner AGW history was reported (*p* < 0.001) because 15.6% (5/32) of the patients’ partners experienced AGWs and 21.9% (7/32) were unsure if they had them, whereas not a single control had partners with AGWs. In addition, more patients than controls had or were unsure about current sexually transmitted infections (two patients reported genital herpes, and one reported chlamydial urethritis).

### 3.2. HPV Infection in Anogenital Warts and Corresponding Hair Samples

A total of 43 DNA samples were obtained from the AGW tissues: 32 at baseline and 11 during follow-up visits. In addition, DNA was extracted from a total of 1232 collected hair samples: 896 from patients and 336 from controls. All the samples tested beta-globin-gene-positive.

As shown in Table 2, all 32 baseline AGW samples exhibited a single Alphapapillomavirus infection, among which 31/32 (96.9%) tested positive for HPV6 or HPV11, and one sample was positive for HPV40. The most frequently observed HPV lineage among the HPV6-positive samples was HPV6 B, with the dominant sublineage being HPV6 B1 (Table 2). All the HPV11-positive AGWs contained sublineage HPV11 A2. No cases of the HPV6 sublineages B4 or B5 or the HPV11 sublineage A1 were found (Table 2).

**Figure 1:**
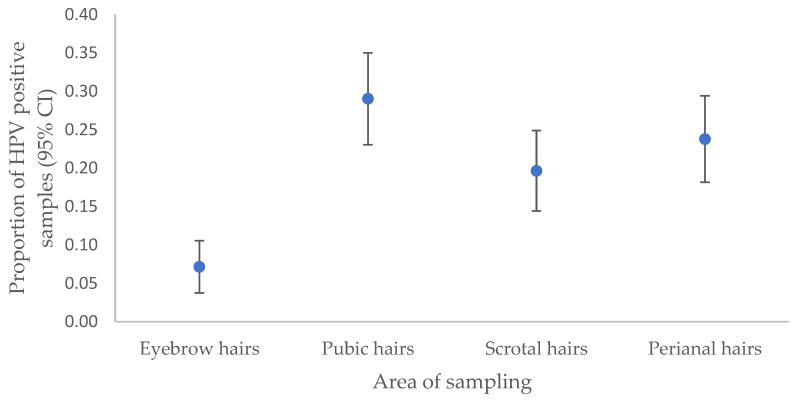
Proportions of HPV6/11-positive hair samples in 32 patients with anogenital warts, collected over the course of the seven study visits (at enrollment and six follow-up visits) by sampling location (eyebrow hairs, pubic hairs, scrotal hairs, and perianal hairs), *n* = 224 for each sampling area; *n* = 896 total number of hair samples tested.

Among all the 896 hair samples collected from the AGW patients during enrollment and six follow-up visits, a total of 178 (19.9%) hair samples tested positive for HPV6 or HPV11. All 28 hair samples collected from the patient with HPV40-related AGWs tested negative for HPV40. At least one HPV6/11-positive hair sample was identified in 31/32 (96.8%) of the patients with AGWs, with a mean of 5.6 HPV6/11-positive hair samples per patient. As shown in Figure 1, the highest prevalence of HPV6/11 was found in pubic hair samples (29.0%; 65/224), followed by perianal hair samples (23.7%; 53/224), scrotal hair samples (19.6%; 44/224), and eyebrow hair samples (7.1%; 16/224).

In contrast to the patients with AGWs, not a single one out of the 336 hair samples collected from the controls tested positive for HPV6/11.

Out of 118 visits at which the patients presented with clinically visible AGWs, in 89 (75.4%) visits, at least one HPV6/11-positive hair sample was found at the same visit. In contrast, out of 100 visits when the patients had no clinically visible AGWs, only in eight (8.0%) visits did the patients have HPV6/11-positive hair sample(s) (*p* < 0.0001).

The proportion of agreement between the presence and absence of HPV6/11 and their lineages and sublineages in AGWs and corresponding hairs exceeded 92% in total. One hundred percent agreement was found in the patients with HPV6 A, HPV6 B2, and HPV6 B3 sublineages; 96.6% in patients with HPV11 and HPV6 B; 96.3% in patients with HPV6 B1; and 90.6% overall in patients with HPV 6. However, all the recorded differences were not statistically significant.

### 3.3. Dynamics of HPV6/11 Infection in Hair Samples

In total, 5 out of 32 patients (15.6%) presented with AGWs only at the baseline visit. In addition to presenting with AGWs at the baseline visit, seven (21.9%) and twenty (62.5%) patients presented with clinically visible AGWs at one and at two or more follow-up visits, respectively.

As shown in Table 3, when analyzing pairs of two consecutive visits for the presence of AGWs in relation to AGW and HPV6/11 status in hair samples at a preceding visit, among the 96/186 pairs of visits when any of the patient’s hair samples tested positive or negative for HPV6/11, 70 (37.6%) and 19 (10.2%) of cases had clinically visible AGWs at their subsequent visit, respectively. A similar pattern was observed with AGW presence at two consecutive visits, whereby 73/185 (39.5%) of cases with and 12 (6.5%) without AGWs also had AGWs at their subsequent visit, respectively. Both associations were highly statistically significant (*p* < 0.0001; Table 3).

Out of the 26/32 patients whose hair samples were initially positive for HPV6/11, 6 (23.1%) remained positive after 11 months (Table 4). At the 11-month mark, significant reductions in the proportion of HPV6/11-positive hair samples were observed for HPV6 (*p* = 0.001), HPV6 lineage B (*p* = 0.001), and HPV6 sublineage B1 (*p* = 0.003).

**Figure 2:**
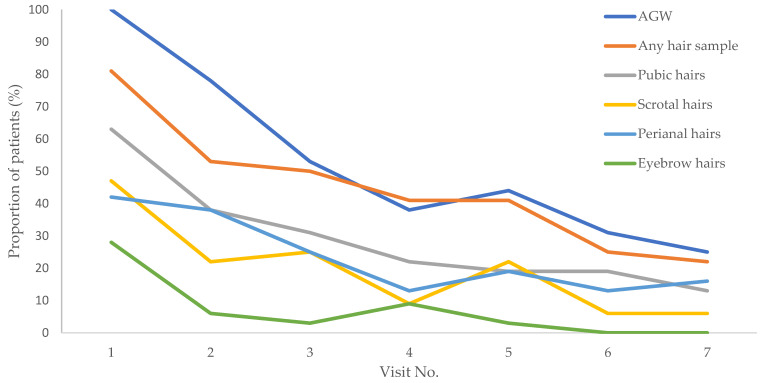
Presence of clinically visible anogenital warts (AGWs) and HPV6/11 positivity rate in collected hair samples through time: at enrollment (visit 1) and during six follow-up visits (visits 2–7). AGW = proportion of patients with clinically visible AGWs during particular visit; any hair sample = proportion of patients with any HPV-positive hair sample collected during a particular visit; pubic hairs, scrotal hairs, perianal hairs, and eyebrow hairs = proportion of patients with an HPV-positive hair sample collected from a certain collection site during a particular visit. Precautions should be taken when interpreting the graph because the time intervals between visits overall and per patient were unequal.

As shown in Figure 2, the proportion of patients with clinically visible AGWs and HPV6/11-positive hair samples decreased over the course of the follow-up visits. Despite fluctuating intervals between visits, we observed similar decreasing trends in the presence of clinically visible AGWs and HPV6/11-positive hair samples; the decreasing trends seem to be similar for all four hair-sampling areas. The likelihood of detecting HPV6/11 in hair samples decreased over time (OR, 0.82 [95% CI, 0.76–0.89], *p* < 0.001). For patients that initially had HPV6-positive AGWs, compared to the other two HPV types (HPV11 and HPV40), no significant differences in the rate of HPV6/11-negative final hair samples were found (OR, 1.05 [95% CI, 0.10–11.08], *p* = 0.97).

The results of the mixed-effects logistic regression model showed that the interaction between time (measured as a continuous variable in months to accommodate unequal time spacing per subject between visits) and hair sampling was not statistically significant. As shown in Table 5, the odds of obtaining HPV6/11-positive samples from eyebrows were statistically significantly lower in comparison to other hair-sampling areas (*p* = 0.034). The odds for HPV-positive hair samples significantly decreased over time (*p* = 0.007; Table 6).

Another mixed-effects logistic regression model with a random intercept was built, from which the interaction effect of the time of follow-up and hair-sample area was omitted and the fixed effects of age, smoking, and shaving of the anogenital region were added (Table 6). In addition to the follow-up time and hair-sampling area, the patients’ age, smoking, and shaving of the anogenital region were statistically significantly associated with HPV6/11 positivity. As shown in Table 6, the odds of obtaining HPV6/11-positive hair samples increased with the patients’ age at enrollment (*p* < 0.001), smoking status (*p* = 0.002), and shaving of the anogenital region (*p* = 0.022).

### 3.4. Recurrence of Anogenital Warts

Out of the thirty-two AGW patients enrolled, two had missing data on AGW presence on some of the intermediate study visits and were excluded from the analysis of AGW recurrence. Of the remaining thirty patients, eight (26.7%) experienced AGW recurrence, defined as the reappearance of AGWs following at least one visit when no AGWs were clinically apparent. As shown in Table 7, no significant differences were found in the AGW recurrence rates across any of the HPV6 and HPV11 lineages and sublineages (*p* > 0.05).

## 4. Discussion

In this prospective study of the dynamics of HPV6/11 infections in plucked anogenital and eyebrow hair samples obtained from 32 men with AGWs undergoing treatment and closely followed for up to 2 years, a close association was seen between the presence of HPV6/11 in hair samples and clinically visible AGWs. The proportion of patients with clinically visible AGWs and HPV6/11-positive hairs declined over the course of the follow-up visits with similar trends, and no particular HPV6/11 genomic variant was linked with an increased AGW recurrence rate; however, sublineage HPV6 B1 showed a significantly higher clearance rate from the hair samples.

All but one baseline AGW sample tested positive for HPV6/11 (31/32, 96.9%), with the predominance of single HPV6 B1 infections accounting for 53.1% of the cases. Previous research also reported the predominance of sublineage HPV6 B1 in European populations and its close association with anogenital infections [25,32,33]. Only a minor share (9.4%) of our patients were infected with sublineage HPV11 A2, which is generally the most commonly detected genomic variant among HPV11 infections worldwide [26,32,34]. In addition, HPV40, commonly detected in AGWs as an HPV6/11 coinfection [7], was detected in this study in a single AGW patient as the only HPV type present.

The predominance of HPV6 B1 infections in our study population prompts further investigation into the evolutionary advantages that this sublineage may possess over other HPV6 genomic variants, as suggested recently [33,35,36]. The HIM (“HPV infection in men”) study, a large prospective study of the natural history of HPV infections in men in three countries (the United States, Mexico, and Brazil), also found an increased risk of AGW development associated with HPV6 B1 genital infections compared to sublineage HPV6 B3 [33]. In addition, the transcriptional activity of the HPV6 B1 long control region (LCR) reference variant was found to be approximately 11 times more active than the HPV6 B3 LCR reference variant [35]. These findings suggest that HPV6 B1 may persist longer as a subclinical infection, thereby contributing to an elevated risk of AGW development. In contrast, the genomic variability of HPV11 appears to be more conserved [26] and less understood, possibly due to substantially fewer HPV11 genomes sequenced from AGWs [34,37].

In this study, there was a high level of agreement (>90%) between HPV6/11 presence and absence as well as the presence of a particular HPV lineage and sublineage in AGWs and corresponding hair samples, suggesting that the identical HPV genomic variant is responsible for HPV persistence in hair samples and subsequent AGW development. These observations are consistent with the findings of our pilot cross-sectional study [20] and align with the outcomes of the HIM study mentioned above, which demonstrated that a genital swab collected prior to the appearance of clinically visible AGWs harbored the identical HPV6 or HPV11 genomic variant as detected in the subsequently developed AGW lesion [33,34].

In this study, the overall prevalence of HPV6/11 infection in 896 hair samples collected from AGW patients during enrollment and six follow-up visits was 19.9%, and out of 336 hair samples collected from the controls, not a single specimen tested positive for HPV6/11. The latter finding contrasts with a previously reported “background” HPV6/11 prevalence in hair samples of apparently healthy “controls,” which ranged from 1.3% to 16.4% [20,21]. Similarly, a 10.4% prevalence of HPV6/11 in subjects without AGWs was found through swabbing anogenital surfaces in the HIM study [38]. The discrepancy in findings could be due to variations in study populations (MSM/MSWM versus MSW), sampling techniques (hairs versus the thorough swabbing of the wide anogenital area), anticontamination measures used during sampling, DNA extraction, and PCR testing, as well as in the HPV-detection methodologies employed.

Some previous studies have labeled hair-plucking samples as “hair follicles” [19,21]. However, such samples include extrafollicular hair shaft segments, potentially carrying HPV DNA from adjacent skin or lesions, and therefore it is difficult to determine whether positive HPV results are solely from the hair follicle or surface contamination of the hair. Therefore, in line with our previous work [20], we refer to such samples as “hair samples” rather than “hair follicles.” Plucked hair may contain intrafollicular HPV, undetectable by swabbing; however, this seems unlikely in productive low-risk HPV infections because intrafollicular keratinocytes are shed outside of the follicle along the growing hair shaft. In addition, swabbing is less uncomfortable for the patient and allows for the sampling of a larger area. This advantage might make swabbing a preferred method in clinical and research settings, balancing scientific accuracy with patient comfort.

The odds of obtaining HPV6/11-positive hair samples in our study increased with two previously established risk factors for genital HPV infection: smoking and shaving of the anogenital region [39], as well as with the patient’s age. The significance of the latter remains to be clarified because it was previously found that, although the burden of genital HPV infections in men remains constant throughout their lifespan, older men achieve clearance of infections faster [40] and are less likely to develop AGWs after a newly acquired HPV infection [41,42].

This study outlines the anatomical distribution of HPV6/11 in hair samples, predominantly in the pubic region as a more reliable site for HPV6/11 detection, followed by the perianal area, scrotum, and eyebrows, similar to our previous findings [20]. In addition, the significantly lower HPV6/11 detection rate in eyebrow hair aligns with the higher susceptibility of anogenital hairs to HPV, given their close proximity to the highly infectious surface of AGWs [19,20,43].

Our patients exhibiting HPV6/11-positive hair samples or clinically visible AGWs at a preceding visit demonstrated substantially increased odds (10- and 11-fold, respectively, *p* < 0.0001 for both) of presenting with clinically visible AGWs at subsequent visits. Interestingly, similar odds were also observed in the HIM study, in which HPV-positive men without prevalent AGWs were nearly 12 times more likely to develop AGWs compared to their HPV-negative counterparts [41]. These findings suggest a similar predictive value of hair sampling, skin swabbing, and historical data of AGWs in forecasting future AGW development. The correlation between AGWs and the presence of HPV6/11 in hair samples is further underscored by the observation that over 95% of our patients had at least one HPV6/11-positive hair sample; in contrast, no HPV6/11 infections were detected in the hair samples obtained from control subjects.

A high share of our 32 patients had more than one AGW episode (defined as a study visit with clinically visible AGWs) because 25 (78.1%) and 20 (62.5%) experienced one and two or more post-initial AGW episodes, respectively. This is substantially more frequent than reported in the HIM study, in which more than one post-initial AGW episode was recorded for only 44% of men [44]. The most likely explanation for the observed difference is the substantially closer longitudinal monitoring of our patients; that is, every 2.6 months (mean) compared to every 6 months in the HIM study. Moreover, the number of AGW episodes in men undergoing treatment might differ according to the standard of care used. In the HIM study, a smaller proportion of men from Brazil experienced multiple AGW events compared to men residing in Mexico and the United States, and this might be partially due to the different standards of care used: in Brazil, excision is the preferred treatment modality, compared with topical treatment in Mexico and the United States [44]. In Slovenia, the preferred treatment for newly diagnosed AGWs is cryotherapy, which was also used in most of our patients.

In this study, AGW recurrence after no visible AGWs for at least 2 months of follow-up was found in 33.3% of patients, reflecting a common proportion of AGW recurrences in other studies [45,46]. No association was found between infections with a particular HPV6/11 genomic variant and AGW recurrence, probably due to the predominance of a single HPV6 genomic variant (sublineage HPV6 B1) and possibly due to the limited number of participants.

Our AGW patients and healthy controls significantly differed in two previously identified risk factors for anogenital HPV infection. More AGW patients than controls reported current STDs or uncertainty about their STD status and a higher incidence of AGWs in their sexual partners, emphasizing the importance of their thorough assessment in clinical settings. Interestingly, some of our patients reported a current chlamydial infection and genital herpes, which were also associated with a prevalent HPV infection in the HIM study [47].

Our study expands the knowledge supporting the potential use of hair samples for various diagnostic purposes. Because the detection of HPV6/11 in hair samples strongly correlates with the presence of wart(s) in the anogenital region, plucked hair samples could be used instead of AGW tissue as a convenient clinical specimen, which is easy to obtain, to reliably diagnose HPV6/11 infections in the anogenital region. Furthermore, the identification and typing of HPV in anogenital hairs could be a valid substitute for diagnosing the AGW-causing HPV type in patients with visible AGWs without testing the tumor tissue itself. This could be particularly beneficial for patients with AGWs who require the determination of the AGW-causing HPV type and who do not consent to the collection of the AGW tissue; in children; in patients where the AGW is in an unfavorable location for collection; in patients favoring self-collection over clinician-collected samples; and in patients who are afraid of pain, emotional distress, and the stigma associated with the collection of AGW tissue or possible complications after collection. Such an indirect diagnosis of the AGW-causing HPV type by testing corresponding anogenital hair samples could be clinically useful in patients who have developed clinically visible AGWs after a complete or incomplete HPV vaccination; this can ensure a noninvasive differential diagnosis in patients suffering from diseases that may clinically resemble AGWs like skin warts, molluscum contagiosum, or Mpox and can be used for various epidemiologic purposes such as the noninvasive impact monitoring of vaccinated cohorts or HPV natural history studies.

Our study has strengths and limitations. The key strength of our study is its prospective design with very frequent patient monitoring, allowing for greater and more detailed insight into the dynamics (HPV persistence, clearance, and recurrence) of HPV6/11 infections and their correlation with AGWs. With a mean interval of 2.6 months between patient visits, to the best of our knowledge, our study is by far the closest longitudinal monitoring of patients with AGWs reported to date. Another strength of our study is that, in contrast to other similar studies, the clinical diagnosis of AGWs was confirmed in all patients by a histological assessment to ensure accurate diagnosis and to avoid the issue of misidentifying other benign skin lesions as AGWs [48]. In addition, when defining the HPV type that causes AGWs, HPV detection was performed in AGW tissue specimens and not from (for example) AGW surface smears, as in the great majority of previous studies. Our approach provides a more precise assessment of the HPV type etiologically linked to AGWs because it allows for the differentiation between wart-causing HPV types and those only colonizing the skin surface, which may not have clinical significance [49,50].

The main limitations of our study are the relatively small number of patients enrolled and nonequal intervals between study visits, which potentially restricted the study’s power to investigate the association(s) between specific HPV6/11 genomic variant(s) and AGW recurrence rates in greater detail. Furthermore, because our study was conducted only on males, the results cannot be generalized to female patients and/or other populations. Finally, an important limitation of our study is the fact that the collected hair samples were tested only for AGW-causing HPV types and not for other HPV types. A longitudinal study of the dynamics of multiple HPV types (including high-risk HPV types) in the hairs of AGW patients would certainly be very interesting, but such an approach would require a different study design and significantly more resources, which were not available to us.

## 5. Conclusions

To provide a better understanding of the natural history of AGWs and the dynamics of HPV6 and HPV11 infection in anogenital hairs and eyebrows, we longitudinally followed a cohort of 32 male patients who were newly diagnosed with AGWs and the same number of age-matched healthy male volunteers for up to 2 years. All the AGW tissue specimens were positive for HPV, with sublineage HPV6 B1 causing 53.1% of the cases. In addition to AGW tissues, 1232 hair samples were prospectively collected. The overall HPV6/11 prevalence in the 896 hair samples collected from the AGW patients was 19.9%, whereas not a single hair sample from the controls (336 hair samples) tested positive for HPV6/11. With a mean of 5.6 HPV6/11-positive hair samples per patient, the highest HPV6/11 prevalence was found in the pubic hair samples (29.0%) and the lowest in the eyebrows (7.1%). The odds of obtaining HPV6/11-positive hair samples increased with smoking, shaving of the anogenital region, and the patients’ age. Our study, which is the closest longitudinal monitoring of patients with AGWs reported to date, showed a close association between the presence of HPV6/11 in hair samples and clinically visible AGWs. The proportion of patients with clinically visible AGWs and HPV6/11-positive hairs declined over the course of six follow-up visits with similar trends. Patients with HPV6/11-positive hairs or clinically visible AGWs at a preceding visit demonstrated substantially increased odds (10- and 11-fold, respectively) of presenting with clinically visible AGWs at a subsequent visit. No particular HPV6/11 genomic variant was linked with an increased AGW recurrence rate, but the sublineage HPV6 B1 showed a significantly higher clearance rate from the hair samples. Due to very close patient monitoring (seven visits every 2.6 months), we demonstrated that, despite treatment (mainly cryotherapy), over 78% and over 62% of patients with newly diagnosed AGWs experience one and two or more postinitial AGW episodes, respectively, which is substantially more frequent than previously reported in the peer-reviewed literature. Our findings underscore the need for further research to provide a more comprehensive understanding of the dynamics of HPV6/11 anogenital infections and the natural history of AGWs, to raise public awareness about AGWs, and to provide free access to gender-neutral HPV vaccinations and vaccinations across wider age groups.

## Figures and Tables

**Table 1:** Sociodemographic characteristics and risk factors for anogenital HPV infection in patients with anogenital warts and controls.

	Controls (*n* = 32)	Patients (*n* = 32)	OR (95%CI)	*p*
Mean age (years) ± SD	30.7 ± 8	30.8 ± 10	1 (0.9; 1.1)	0.955
≥14 years of education	12 (37.5)	13 (40.6)	1.1 (0.4; 3.1)	0.798
Currently employed	21 (65.6)	20 (62.5)	0.9 (0.3; 2.4)	0.795
Marital status				
Married	7 (21.9)	4 (12.5)	1	
Cohabiting	10 (31.3)	15 (46.9)	2.6 (0.6; 11.4)	0.197
Single	15 (46.9)	13 (40.6)	1.5 (0.4; 6.4)	0.570
Cigarette smoking	14 (43.8)	19 (59.4)	1.9 (0.7; 5.1)	0.213
History of skin disease				
No	20 (62.5)	15 (46.9)	1	
Yes	7 (21.9)	14 (43.8)	2.7 (0.9; 8.2)	0.088
Unsure	5 (15.6)	3 (9.4)	0.8 (0.2; 3.9)	0.782
Current STI other than AGWs				**0.034** *
No	29 (90.6)	22 (68.8)		
Yes	0	3 (9.4)		
Unsure	3 (9.4)	7 (21.9)		
Past STI				0.329
No	30 (93.8)	27 (84.4)		
Yes	2 (6.3)	4 (12.5)		
Unsure	0	1 (3.1)		
Ever tested for STI	8 (25)	8 (25)	1 (0.3; 3.1)	1
Mean age at first sexual intercourse (years) ± SD	17.2 ± 2.2	17.7 ± 2	1.1 (0.9; 1.4)	0.373
Currently sexually active	26 (81.3)	23 (71.9)	0.6 (0.2; 1.9)	0.379
Partners with present history of AGWs				
No	32 (100)	20 (62.5)		**<0.001** *
Yes	0	5 (15.6)		
Unsure	0	7 (21.9)		
Lifetime no. sexual partners				
1	6 (18.8)	3 (9.4)	1	
≤5	6 (18.8)	8 (25)	2.7 (0.5; 15.3)	0.270
6–10	9 (28.1)	10 (31.3)	2.2 (0.4; 11.6)	0.344
>10	11 (34.4)	11 (34.4)	2 (0.4; 10.1)	0.401
No. sexual partners in past year				0.202 *
0	0	3 (9.4)		
1	22 (68.8)	18 (56.3)		
1–5	8 (25)	9 (28.1)		
>5	2 (6.3)	2 (6.3)		
Sexual orientation				0.220 *
MSW	30 (93.7)	27 (84.4)		
MSM	2 (6.3)	3 (9.4)		
MSWM	0	2 (6.3)		
Condom use				
Never	4 (12.5)	4 (12.5)	1	
Occasionally	22 (68.8)	24 (75)	1.1 (0.2; 4.9)	0.910
Always	6 (18.8)	4 (12.5)	0.7 (0.1; 4.4)	0.672
Circumcised	2 (6.7)	8 (25)	4.7 (0.9; 24.1)	0.066
Shaving of anogenital region	18 (58.1)	18 (56.3)	0.9 (0.3; 2.5)	0.884

Data are presented as *n* (%) unless otherwise indicated. Abbreviations: OR = odds ratio; 95% CI = 95% confidence interval; *p* = *p* value; *SD* = standard deviation; AGW = anogenital warts; STI = sexually transmitted infection; MSW = men who have sex only with women; MSM = men who have sex only with men; MSWM = men who have sex with women and men; * = likelihood ratio test. Statistically significant associations (*p* < 0.05) are shown in bold.

**Table 2:** Distribution of HPV types, lineages, and sublineages in baseline anogenital wart tissue samples of the 32 patients.

HPV Type, Lineage, and Sublineage	No. (%) Samples
**HPV6**	28 (87.5)
HPV6 A *	2 (7.1)
HPV6 B	26 (92.9)
HPV6 B1	17 (65.4)
HPV6 B2	5 (19.2)
HPV6 B3	2 (7.7)
HPV6 B untypable	2 (7.7)
**HPV11**	3 (9.4)
HPV11A2	3 (100.0)
**HPV40**	1 (3.1)

* HPV6 sublineage could not be determined in both HPV6 lineage A-positive samples.

**Table 3:** Presence of clinically visible anogenital warts (AGWs) in relation to HPV6/11 in hair samples and AGWs at a preceding visit. For AGWs vs. HPV in hair samples at a preceding visit, *n* = 186. For AGWs vs. AGWs at a preceding visit, *n* = 185.

Previous Visit	AGWs				OR (95% CI)	*p*
	YES		NO			
	*n*	%	*n*	%		
Hairs YES	70	37.6	26	14.0	10.06 (5.11–19.8)	**<0.0001**
Hairs NO	19	10.2	71	38.2		
AGWs YES	73	39.5	35	18.9	11.30 (5.41–23.58)	**<0.0001**
AGWs NO	12	6.5	65	35.1		

Abbreviations: OR = odds ratio; 95% CI = 95% confidence interval; *p* = *p* value; Hairs YES = HPV6/11 detected in at least one hair sample at a preceding visit; Hairs NO = no HPV6/11 detected in hair at a preceding visit; AGW YES = presence of clinically visible AGW at a preceding visit; AGW NO = no clinically visible AGW at a preceding visit. Statistically significant associations (*p* < 0.05) are shown in bold.

**Table 4:** Number and proportion (%) of participants with HPV infection in hair samples by HPV lineage at enrollment in the study and after 11 months of follow-up.

	Baseline	After 11 Months *		*p*
		No	Yes	
HPV6	No	5 (62.5)	3 (37.5)	**0.001**
	Yes	19 (79.2)	5 (20.8)	
HPV 6 A	No	29 (100)	0 (0)	0.48
	Yes	2 (100)	0 (0)	
HPV 6 B	No	7 (77.8)	2 (22.2)	**0.001**
	Yes	17 (77.3)	5 (22.7)	
HPV 6 B1	No	13 (92.9)	1 (7.1)	**0.003**
	Yes	12 (80)	3 (20)	
HPV 6 B2	No	25 (96.2)	1 (3.8)	1
	Yes	1 (33.3)	2 (66.7)	
HPV 6 B3	No	27 (100)	0 (0)	0.48
	Yes	2 (100)	0 (0)	
HPV 11 A2	No	30 (100)	0 (0)	1
	Yes	1 (50)	1 (50)	

* Due to unequal time intervals between patient follow-up visits, the cross-sectional time point was analyzed for which follow-up data were available for all 32 patients enrolled with AGWs (11 months). Statistically significant associations (*p* < 0.05) are shown in bold.

**Table 5:** Association between follow-up time, hair-sampling area, their interaction, and HPV positivity (results of mixed model logistic regression analysis).

Hair-Sampling Area/Follow-Up Time	OR (95% CI)	*p*
Perianal region	1	
Eyebrows	0.36 (0.14; 0.92)	**0.034**
Pubis	1.48 (0.68; 3.25)	0.324
Scrotum	0.83 (0.38; 1.82)	0.634
Follow-up time	0.86 (0.78; 0.96)	**0.007**
Perianal region	1	
Eyebrows	0.81 (0.65; 1.00)	0.055
Pubis	0.99 (0.89; 1.11)	0.874
Scrotum	0.97 (0.88; 1.07)	0.566

Abbreviations: OR = odds ratio; 95% CI = 95% confidence interval; *p* = *p* value. Statistically significant associations (*p* < 0.05) are shown in bold.

**Table 6:** Association between patients’ age at enrollment, smoking, shaving of the anogenital area, and presence of HPV6/11 in collected hairs. Lifestyle factors included participants’ age at enrollment, smoking status, and shaving of the anogenital region.

Hair-Sampling Area/Age/Lifestyle Factors	OR (95% CI)	*p*
Perianal hairs	1	
Eyebrow hairs	0.17 (0.08–0.34)	**<0.001**
Pubic hairs	1.42 (0.68–2.98)	0.354
Scrotal hairs	0.71 (0.37–1.34)	0.29
Follow-up time	0.84 (0.78–0.91)	**<0.001**
Patients’ age at enrollment	1.06 (1.04–1.08)	**<0.001**
Noncigarette smoking	1	
Cigarette smoking	3.04 (1.49–6.22)	**0.002**
Shaving—no	1	
Shaving—yes	2.34 (1.13–4.82)	**0.022**

Abbreviations: OR = odds ratio; 95% CI = 95% confidence interval; *p* = *p* value. Statistically significant associations (*p* < 0.05) are shown in bold.

**Table 7:** Anogenital wart recurrences (AGW) by HPV lineages and sublineages.

HPV Type, Lineage, and Sublineage	Baseline	AGW Recurrence		*p*
		No (*n* (%))	Yes (*n* (%))	
HPV6	No	1 (4.5)	0 (0)	0.425
	Yes	21 (95.5)	8 (100)	
HPV6 A	No	18 (90.0)	7 (100)	0.263
	Yes	2 (10.0)	0 (0)	
HPV6 B	No	3 (15.0)	0 (0)	0.165
	Yes	17 (85.0)	7 (100)	
HPV6 B1	No	8 (42.1)	2 (33.3)	0.700
	Yes	11 (57.9)	4 (66.7)	
HPV6 B2	No	16 (84.2)	4 (66.7)	0.369
	Yes	3 (15.8)	2 (33.3)	
HPV6 B3	No	17 (89.5)	6 (100)	0.283
	Yes	2 (10.5)	0 (0)	
HPV11 A2 *	No	20 (90.9)	7 (87.5)	0.787
	Yes	2 (9.1)	1 (12.5)	
HPV40 **	Yes	1 (100)	0 (0)	—

* All HPV11-positive AGW samples harbored sublineage HPV11 A2. ** Because only a single AGW patient tested positive for HPV40, the association between the presence of this HPV type and AGW recurrence was not tested.

## Data Availability

The vast majority of the generated data are presented in the manuscript. Further data generated in this study are available upon request from the corresponding author.
